# CD19^ (+)^ CD56^ (–)^ myeloma arising in a patient who failed two courses of immunosupressive therapy for aplastic anaemia

**DOI:** 10.3332/ecancer.2017.720

**Published:** 2017-02-14

**Authors:** Nigel P Murray, M Amparo Ruiz, G Maximiliano Miranda

**Affiliations:** 1 Service of Medicine, Hospital Carabineros of Chile, Nuñoa, 7770199 Santiago, Chile; 2 Faculty of Medicine, University Finis Terrae, Providencia, 7501015 Santiago, Chile; 3 Service of Pathology, Hospital Carabineros of Chile, Nuñoa, 7770199 Santiago, Chile

**Keywords:** aplastic anemia, myeloma, immunosuppressive therapy

## Abstract

Patients diagnosed with severe aplastic anaemia and without a compatible bone marrow transplant donor are treated with immunosuppressive therapy. These patients are found with time to develop a clonal disease such as myelodysplasia or paroxysmal nocturnal haemoglobinuria. However, the development of plasma cell dyscrasias is rare. We report the case here of a patient treated with immunosuppressive therapy who went on to develop myeloma 11 months after being diagnosed with severe aplastic anaemia. We include here a review of the literature.

## Introduction

In patients without a compatible sibling or unrelated donor standard immunosuppressive therapy (IT) with anti-thymocyte globulin (ATG) and cyclosporine A (CyA) produces excellent results with a reported response rate of 58–77% and a survival rate of 58% at 11 years [1–4]). The most serious complication of IT is the development of clonal disease, including the development of myelodysplasia and expansion of paroxysmal nocturnal haemoglobinuria (PNH) clones.

Immunophenotypic analysis with anti-CD19 and anti-CD56 can clearly distinguish between normal plasma cells and myeloma cells. Normal plasma cells are CD19 (+) CD56 (-), whereas in myeloma approximately 65% are CD19 (-) CD56 (+), 30% CD19 (-) CD56 (-) and 5% CD19 (+) CD56 (+) [5, 6]. We present a case of myeloma expressing the rare phenotype CD19 (+) CD56 (-) in a 15-year-old female patient occurring six months after failing to respond to a second cycle of ATG-methylprednisone-cyclosporine for aplastic anaemia.

## Clinical case

A 15-year-old girl presented with a three month history of extensive bruising and hypermenorrhea of up to 18 days. A full blood count showed a haemoglobin of 3.5 g/dL mean corpuscular volume of 111.6 fL, platelets of 6000/mm^3^, white cell count of 1500/mm^3^, an absolute neutrophil count of 450/mm^3^, and reticulocyte count of 0.5%. A provisional diagnosis of aplastic anaemia was made, and the supplementary testing showed; tests for hepatitis A, B, C, and HIV negative, antinuclear, anti-DNA antibodies negative, negative for Epstein-Barr virus and cytomegalovirus. Immunoglobulin (Ig) A 77 mg/dL (normal range 70–400), IgG 773 mg/dL (normal range 700–1600), and IgM 54 mg/dL (40–230). CD55/CD59 expression normal, bilirubin 0.37 (normal range <1.0), ferritin 31 (normal range 10–150), and lactate dehydrogenase 209 IU (normal range 100–250).

Bone marrow biopsy showed less than 20% cellularity. There were immature and precursor cells of the erythroid and myeloid series in central accumulations and paratrabecular location with very few mature forms. No megakaryocytes were observed.

**Figure 1.**
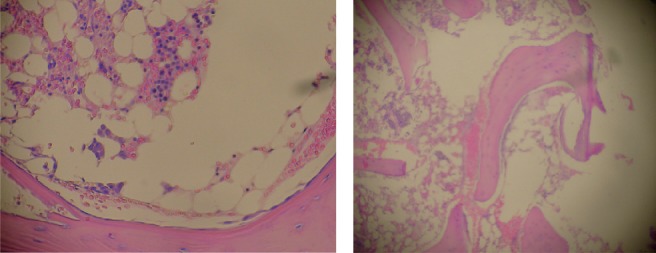
Bone marrow biopsy (H & E), 20% cellularity (magnification x 100).

Staining for myeloperoxidase showed positivity in 30% of immature cells which were arranged as paratrabecular sheets, while CD34 staining cells were less than 5%, with a central focus of immature cells staining positive for both CD34 and myeloperoxidase. Staining for PNCA was less than 1% and there was no evidence of fibrosis or lymphoid follicular formation (Figure 1). Bone marrow cytogenetics revealed a normal 46XX karyotype.

A diagnosis of aplastic anaemia was made. A suitable bone marrow donor was unavailable and hence immunosuppressive therapy with anti–thymocyte globulin, methylprednisone, and cyclosporine was started. There was no response at six months and a second cycle was repeated, again failing to achieve a response.

Eleven months after the initial presentation, the patient presented with high fever of 40ºC, and bilateral cervical adenopathy without any other localising signs. A CT scan of the neck showed bilateral cervical and jugular chain adenopathy of up to 22 mm with necrosis. Empirical antibiotic therapy was started with amikacin and the combination antibiotic piperacillin–tazobactam with platelet support (hydrocortisone and chlorpheniramine prophylaxis was administered with platelet transfusions). Blood and urine cultures were persistently negative. With the adenopathy increasing in size and to exclude a lymphoproliferative disease, an excisional biopsy of the node was performed along with a repeat bone marrow biopsy.

**Figure 2.**
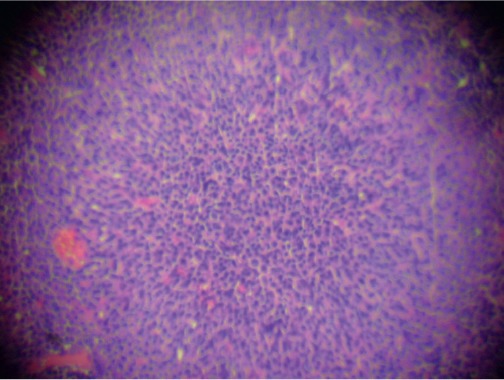
Low power biopsy ganglia (x 10).

**Figure 3.**
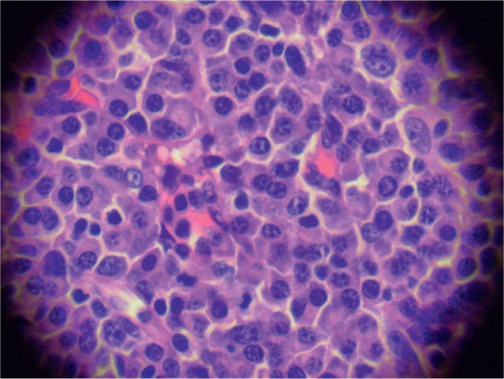
Infiltration by plasma cells (x 400).

The biopsy of the node showed a conserved structure but with loss of the lymphoid follicles. There was 70% of the lymph node parenchyma being replaced with an infiltration of morphologically mature plasma cells as well as loss of the normal architecture (Figure 2, 3).

**Figure 4.**
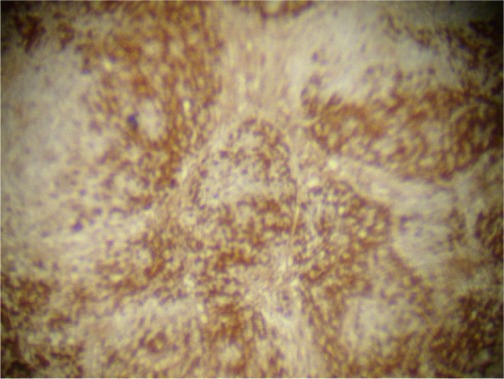
Plasma cells CD38 (+) (x 10).

**Figure 5.**
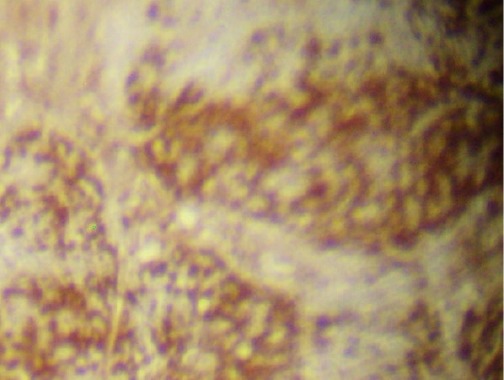
Plasma cells CD19 (+) (x 10).

**Figure 6.**
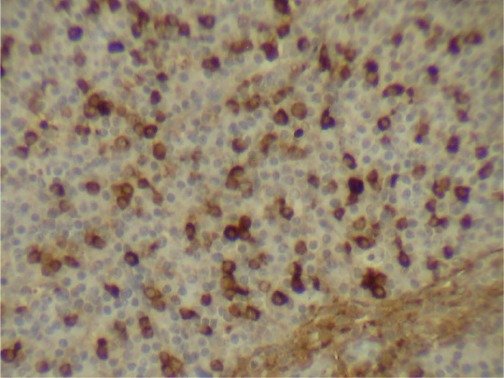
Kappa expression (x 400).

**Figure 7.**
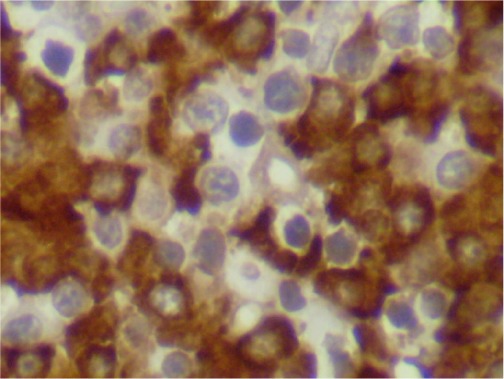
Lambda expression (x 600).

The infiltrating cells were CD38 positive (Figure 4) and CD19 positive (Figure 5), with a kappa–lambda ratio of 1:4 (Figure 6–7)

**Figure 8.**
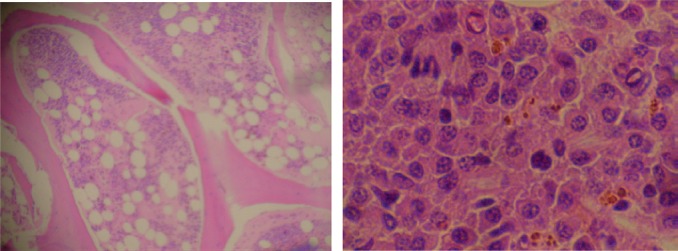
Bone marrow biopsy showing hypercellular features and infiltration by plasma cells (x 400).

**Figure 9.**
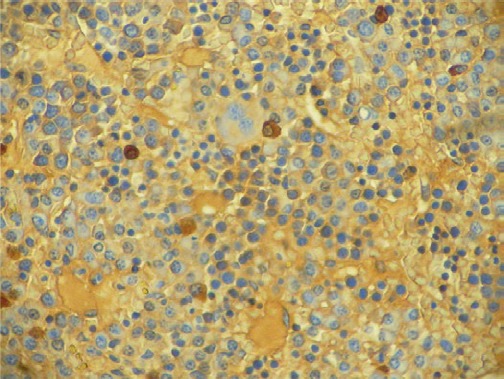
Bone marrow biopsy stained for kappa (x 400).

**Figure 10.**
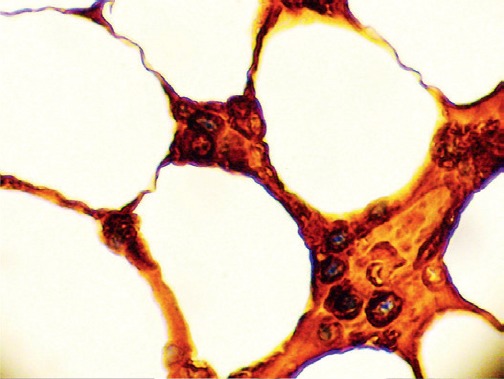
Bone marrow stained for lambda (x 1000).

**Figure 11.**
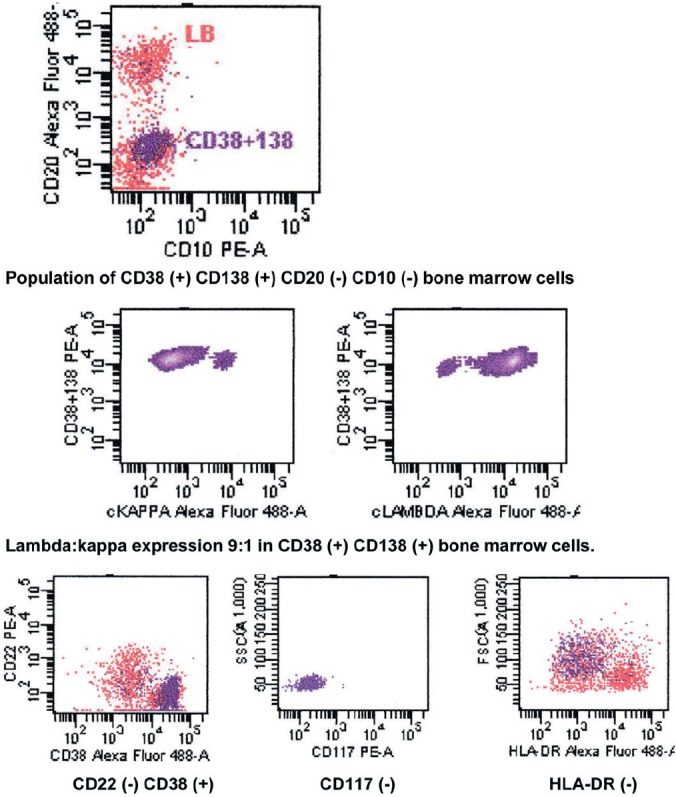
Flow cytometry of bone marrow showing CD56 (-) CD19 (+) phenotype.

The bone marrow biopsy (Figure 8) showed a variable cellularity occupying between 10–90% of the intertrabecular spaces with over 70% being plasma cells, diffusely infiltrated between a few myeloid precursors. Immunocytochemistry showed a lambda:kappa ratio of 9:1 (Figure 9, 10), the plasma cells being negative for CD20 and positive for CD19, CD38, and CD138, and 20% of plasma cells being positive for Bcl-2. Flow cytometry of the bone marrow showed that 23% of the nucleated cells expressed CD45, CD38i, CD138m, CD19, and cytoplasmic lambda light chain. The cells were CD56, CD117, CD20, CD22, and HLA–DR negative (Figure 11).

There was a hyper–gammopathy of 10 g/dL (normal range 3.0–4.5), with an IgA lambda monoclonal protein of 4.20 g/dL, total IgG 535m g/dL, and total IgM 32 mg/dL, both IgG and IgM being decreased, and a positive Bence-Jones protein. Serum creatine and calcium levels were normal and a skeletal survey showed a few small osteolytic lesions in the lateral skull x–ray.

A diagnosis of plasma cell dyscrasia consistent with myeloma was made. Because of the severe cytopaenia, dexamethasone 40 mg days 1–4 with a 10 day cycle was started. After six cycles, the Bence-Jones protein was undetectable, and the monoclonal protein had decreased to 2.30 g/dL. However, the patient presented with septic shock and died.

## Discussion

There are only a few cases in the literature of aplastic anaemia progressing to myeloma. Manoharan *et al* reported a case in 1981 [7], and a second case of acquired aplastic anaemia evolving into myelodysplasia and finally myeloma was reported in 1991 [8].

There are cases reported of aplastic anaemia associated with a minimal serum M–protein [9] or a marked reactive plasmacytosis secondary to a drug reaction [10], autoimmune disease [11], or infection [12].

In the case we describe here, we consider from the initial findings that myeloma was not present. Initial bone marrow biopsy revealed no infiltration with plasma cells (with we recognising the fact that myeloma can also present with severe marrow hypoplasia) [13]. There was no serum paraprotein, and the patient had normal immunoglobulin levels. There was no evidence of a hypoplastic myelodysplasia at the initial biopsy as suggested by the low PNCA and CD34 expression [14]. The clinical and pathological findings were consistent with acquired aplastic anaemia.

After IT failure, the patient was considered to have developed myeloma based on the 2015 NCCN guidelines. Sahara *et al* [15] first reported a case of CD19 (+) CD56 (–) myeloma. The CD38, CD138 phenotype combined with low levels of CD45 expression, excluded the possible contamination with CD19 (+) CD56 (–) lymphoid cells. The cellular morphology, monoclonal plasma cell proliferation, monoclonal gammopathy, Bence-Jones protein, and osteolytic lesions supported the diagnosis of myeloma. Although the cervical lymph nodes were infiltrated by plasma cells with the same immunophenotype as the bone marrow, this was considered to be extra–medullar extension rather than a plasmacytoma.

Studies have reported that low CD56 expression is associated with extensive bone marrow and blood involvement; a more aggressive disease and a significantly shorter survival [16]. It has been recommended that CD56 (–) myeloma be treated intensively to overcome this poor response [17]. However, treatment options were limited because of the severely hypocellular bone marrow, dexamethasone produced an initial response with decreases in the M-protein and disappearance of the Bence-Jones protein. Although showing a response, the patient died as a result of septic shock and acute multiorgan failure.

The differential diagnosis of plasmablastic lymphoma was also considered. However, the lesion being lytic with bone marrow involvement, having morphological characteristics of myeloma rather than a large cell lymphoma, having a monoclonal paraprotein with a positive Bence-Jones protein, and finally being HIV negative, all supported the diagnosis of myeloma rather than lymphoma. Plasmablastic lymphoma is an uncommon diffuse large B–cell lymphoma with a predilection for individuals HIV positive or with immunosuppression. Biopsy of the lymph node in plasmablastic lymphoma shows poorly differentiated plasma cells with low or absent B–cell markers (CD19, CD20, and CD22, and surface/cytoplasmic immunoglobulin), and positivity for plasma cell markers such as CD38 and CD138 [16].

## Conclusion

In summary, our case presents the rare CD19 (+) CD56 (–) phenotype of myeloma arising in a patient treated by IT for aplastic anaemia. Treatment of what is considered to be an aggressive form of myeloma is severely limited in patients with poor marrow reserve and lack of a transplant donor. With only a few cases reported of myeloma arising from aplastic anaemia treated with IT reported in the literature, it is difficult to confirm this association. However, in this case report there did not appear to be evidence of myeloma pre-IT whereas 18 months after the initial diagnosis and two cycles of IT, the evidence suggested the rare phenotype of myeloma.

## Ethical considerations

This case study was approved by the Hospital de Carabineros Ethical Committee, complying with the Chilean Law 20,458 of patients’ rights.

## Conflicts of interest

The authors report no conflicts of interest.
